# A Real-Time Terahertz Time-Domain Polarization Analyzer with 80-MHz Repetition-Rate Femtosecond Laser Pulses

**DOI:** 10.3390/s130303299

**Published:** 2013-03-11

**Authors:** Shinichi Watanabe, Naoya Yasumatsu, Kenichi Oguchi, Masatoshi Takeda, Takeshi Suzuki, Takehiro Tachizaki

**Affiliations:** Department of Physics, Faculty of Science and Technology, Keio University, 3-14-1 Hiyoshi, Kohoku-ku, Yokohama, Kanagawa 223-8522, Japan; E-Mails: yasumatsu@wlab.phys.keio.ac.jp (N.Y.); oguchi@wlab.phys.keio.ac.jp (K.O.); takeda@wlab.phys.keio.ac.jp (M.T.); suzuki@wlab.phys.keio.ac.jp (T.S.); tachi@phys.keio.ac.jp (T.T.)

**Keywords:** terahertz spectroscopy, polarization analysis, electro-optic sensor

## Abstract

We have developed a real-time terahertz time-domain polarization analyzer by using 80-MHz repetition-rate femtosecond laser pulses. Our technique is based on the spinning electro-optic sensor method, which we recently proposed and demonstrated by using a regenerative amplifier laser system; here we improve the detection scheme in order to be able to use it with a femtosecond laser oscillator with laser pulses of a much higher repetition rate. This improvement brings great advantages for realizing broadband, compact and stable real-time terahertz time-domain polarization measurement systems for scientific and industrial applications.

## 1. Introduction

Optical polarization sensing and spectroscopy are fundamental experimental tools in materials science. The development of highly sensitive and ultrafast polarization measurement technologies in the visible wavelength range have enabled the study of various basic properties of materials, a few of which are dielectric constants, measured by performing ellipsometry [1]; ultrafast magnetization dynamics, characterized by Faraday/Kerr rotation spectroscopy using ultrafast laser pulses [2–5] and the conformation dynamics of proteins, obtained with a circular dichroism measurement [6].

Polarization spectroscopy and ellipsometry in the terahertz frequency range with a much lower photon energy of several meV have become a challenging research area in last two decades [7–10]. These techniques have become important tools for investigating low-energy dynamical phenomena in various kinds of materials such as molecules [11,12], superconductors [13–17], multiferroics [18], two-dimensional electron gases [19], graphene [20], magnetic materials [21–25] and topological insulators [26,27]. They are also important in many fields of applied science for investigating carrier density and mobility in semiconductors [8,28–30]; local stress and optical axis, probed by terahertz birefringence measurements [31–34] and physiological conditions, probed by reflection polarimetry of human skin [35,36]. In addition, terahertz time-domain polarimetry, which allows us to extract the instantaneous direction of the electric-field (E-field) vectors within a single-cycle of the electromagnetic oscillation, opens new avenues for investigating the surface topography of materials [37] and for separate determination of the optical Faraday and Kerr rotation angles using echo signals [26], a result that cannot be attained with the conventional polarization measurement technique for much higher frequencies.

Development of the polarization sensing technology for the terahertz frequency range has long been pursued by the strategy of inventing new types of terahertz polarizers with wider frequency coverage and a better extinction ratio, where both requirements are quite important for basic characterization of materials, though it is quite difficult to satisfy both conditions. Recent remarkable progress in this area includes achieving an extinction ratio of 84.9 dB [38], developing a flexible polarizer using nanoimprint technology [39], creation of a circular polarizer [40]. There have been continuous efforts to understand the basic electromagnetic properties in metamaterial structures [41–50] and to realize their potential practical applications [51,52], which is necessary to design such new terahertz polarization devices. Terahertz polarizers using an array of carbon nanotubes have also been proposed and demonstrated [53,54]. Several methods to characterize the state of polarization of the terahertz waves by performing terahertz time-domain spectroscopy using the polarizers are proposed and demonstrated [55–57].

Another approach to polarization-sensing technologies in the terahertz frequency range that avoids using the terahertz polarizers is to utilize a semiconductor photoconductive antenna device with two or three gaps that have different polarization sensitivities [7,58–61]. Helicity-sensitive terahertz sensors using field-effect transistors have also been reported [62]. In a terahertz time-domain spectroscopy (THz-TDS) measurement using the electro-optic (EO) sampling method, one can use the EO crystal as a “natural” polarizer having a terahertz E-field sensitivity that depends on the relative direction between the crystal axes and the polarization direction of the terahertz waves [63–68]. These experimental schemes have the advantage of realizing broadband polarization measurements that are not limited by the bandwidth of the terahertz polarizers.

In order to improve the precision and the speed of determining the polarization direction, modulation techniques are used. The commonly used technique is to rapidly rotate the wire-grid polarizer or a waveplate in the terahertz frequency region to modulate the signal, and then determine the polarization direction by extracting the modulated components of the signal [69–71]. We recently proposed and demonstrated a new experimental scheme for the terahertz polarization measurement involving rapidly rotating the EO crystal [72] to modulate the EO signal; this is much easier to achieve than it would be with the wire-grid counterpart since the EO crystal is much smaller than the wire-grid polarizers and it is quite easy to achieve a stable rotation with a frequency up to 100 Hz (6,000 rotations per minute). We showed that a mechanical rotation of the <110>-oriented zinc-blend crystal with an angular frequency ω adds two frequency components, ω and 3 ω, to the EO signal, and the analysis of their phases makes it possible to quickly and precisely determine the polarization direction of the electric-field vector of the terahertz waves [72]. Our method is based on the conventional THz-TDS measurement systems [73] so that we can easily perform the time-dependent waveform analysis of the terahertz E-field vectors that includes the polarization information.

Quite importantly, our modulation technique does not require wire-grid polarizers which sometimes restrict the measureable frequency range of the polarization-sensitive terahertz spectroscopy. Instead, we use the <110>-oriented zinc-blende crystal as a natural polarizer, and thus we may easily extend our polarization-resolved terahertz waveform analysis of up to about 60 THz if we use ultrashort femtosecond laser pulses with a pulse width of less than 10 fs as a light source and choose proper zinc-blende crystals [74,75]. In [72], we demonstrated this method by using a regenerative amplifier laser system with a pulse width of about 150 fs as a light source for both the terahertz pulse generation and detection. It is quite desirable to achieve the same measurement scheme by using a laser oscillator system with a higher repetition rate, because one can commercially obtain oscillator systems with an extremely short (<10 fs) pulse width, which opens a new horizon on the ultra-broadband polarization-resolved terahertz spectroscopy. A stableness and compactness are the additional advantages to use the oscillator systems which are very useful in practical usage.

In principle, our method can also be applied to the THz-TDS systems with the oscillator system with a higher repetition rate, however, the terahertz E-field magnitude and thus the EO signal are very weak in this case, and a careful design of the measurement system should be required. In this paper, we demonstrate a terahertz polarization measurement system with the spinning EO sensor method by using 80-MHz repetition-rate Ti:Sapphire femtosecond laser pulses with a pulse width of about 100 fs, and we describe the detailed methodology for achieving the real-time and precise polarization measurement. We believe that one can further apply the present technique to the broadband terahertz spectroscopy by using a femtosecond laser pulse with much shorter pulse width in future and thus this paper brings great advantages for realizing broadband, compact and stable real-time terahertz time-domain polarization measurement systems, which should accelerate the scientific and industrial applications.

## 2. Methodology

**Figure 1.**
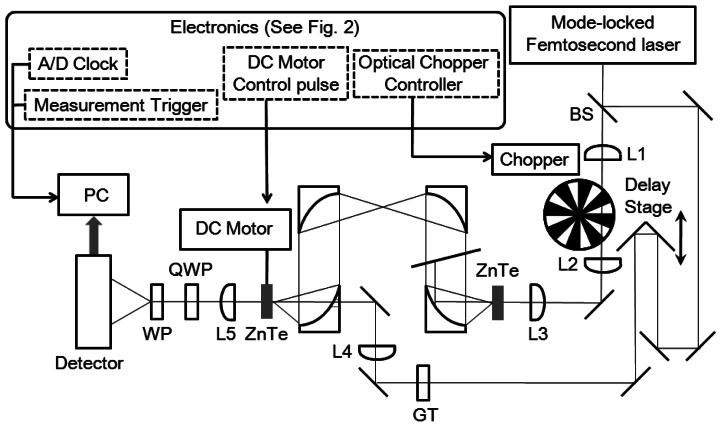
Experimental setup of the measurements of the magnitude and the polarization direction of the terahertz waves with a spinning electro-optic (EO) sensor method with 80-MHz repetition-rate femtosecond laser pulses. Electronic pulse signals used to control the mechanical components and the analog-to-digital converter are also indicated by the words inside the boxes with dotted line borders. The polarization of the probe laser pulse is set parallel to the *x*-axis which is defined as the direction perpendicular to the propagation direction of the probe laser pulse and parallel to the sheet. The polarization of the pump laser pulse used to generate terahertz waves by optical rectification in the (110)-oriented ZnTe EO crystal is set perpendicular to the *x*-axis. BS: a beam sampler, L1–L5: optical lenses, Chopper: a mechanical optical chopper, GT: a Glan-Thompson prism, PM1–PM4: parabolic mirrors, QWP: a quarter-wave plate and WP: a Wollaston prism.

**Figure 2.**
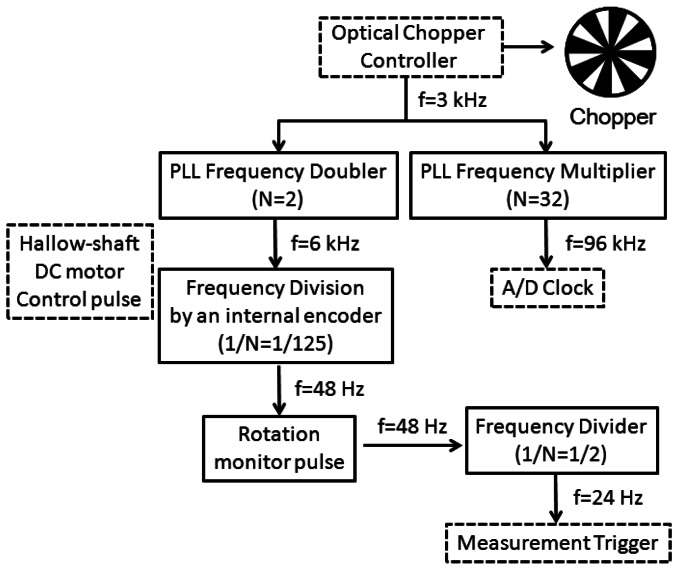
Block diagram of the instrument electronics. Electronic pulse signals used to control the instruments are indicated by the words in the boxes bordered by dotted lines, as in Figure 1.

**Figure 3.**
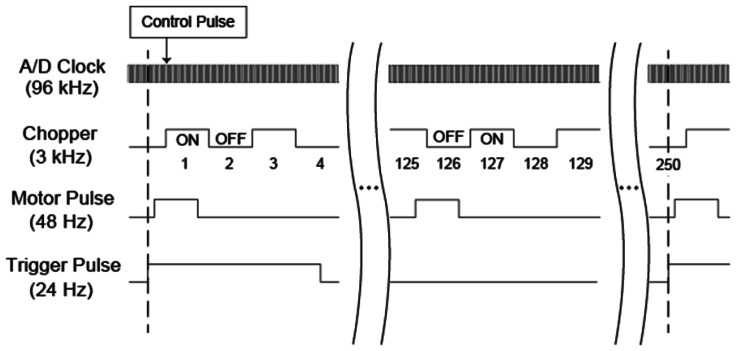
Timing chart of the measurement system. The times to begin the single-cycle measurements are indicated by the two dotted lines. In practice, one of the two dotted lines cannot be used to start the measurement, because one needs additional time for the numerical computations. Four clock pulses are matched in phase with each other with a timing jitter of about 2 μs, while their rising-edge timings are different. The frequency of each electronic pulse is also indicated.

In this section, we describe a detailed methodology for achieving a terahertz polarization measurement by the spinning EO sensor method by using 80-MHz repetition-rate femtosecond laser pulses. In particular, we describe the electronic part of the system in detail, which is crucial for achieving a stable measurement with a high signal-to-noise ratio. A schematic of the system is shown in Figure 1. We use a femtosecond near-infrared (IR) laser oscillator with a wavelength of 800 nm, a repetition rate of 80 MHz, a pulse width of about 100 fs, and a power of 1 W for both the terahertz pulse generation and detection. The setup is almost the same as the conventional THz-TDS systems with an EO sampling method [73] except for the spinning EO crystal attached to a stabilized hollow shaft motor that rotates with a frequency of 48 Hz. The pulse energy of the near-IR laser pulse for THz pulse generation is typically 9 nJ just before the ZnTe crystal, while that of the probe laser pulse just before the spinning ZnTe crystal is 0.07 nJ. A mechanical chopper is required in the near-IR beam path of the terahertz pulse generation scheme in order to perform a signal subtraction algorithm as we describe below. The blades of the chopper have a 1:1 mark:space (duty cycle) ratio. We place the chopper between two optical lenses in order to make the spot size of the laser beam as small as possible when it passes through the chopper, which is important to improve the on/off modulation depth of the intensity of the terahertz pulse with a square pulse sequence. The on/off modulation frequency is typically 3 kHz, and we use this frequency as a master clock to govern the timing of all parts of the measurement system (see also Figures 2 and 3). The EO detection of the terahertz E-field magnitude is performed by measuring the ellipticity of the optical probe pulse induced by the Pockels effect in the crystal. When the terahertz E-field magnitude is not so large, the intensity difference signal Δ*I* between the *x*- and *y*-polarized components of the probe pulse after passing through the <110>-oriented zinc-blend crystal and a quarter-wave plate is proportional to the terahertz E-field magnitude. In our measurement setup, to measure Δ*I*, we use a homemade detector including a current-to-voltage converter with a low-pass filter in its analog electronic circuit with a cut-off frequency of about 100 kHz, so that the high-frequency (80 MHz) component of the EO signal is attenuated.

In [72], we found that if we rotate the <110>-oriented EO crystal with an angular frequency of *ω*, the time-dependent intensity difference signal Δ*I*(*T*) becomes:

(1) $$\Delta I\left(T\right)\propto E_{a}\left\{\frac{1}{2}\text{cos}\left(\omega T+\beta_{0}+\gamma \right)+\frac{3}{2}\text{cos}\left(3\omega T+3\beta_{0}-\gamma \right)\right\}$$

where the *x*-direction is defined as the polarization direction of the optical probe pulse, *E_a_* is the magnitude of the terahertz electric field pulses, *β*_0_ is the initial angle (at *T* = 0) between the *x*-axis and the [1̅10] direction of the rotating EO crystal and γ is the angle between the *x*-axis and the polarization direction of the terahertz waves that we want to measure. The magnitude and phase information of Equation (1) can be used to determine the E-field magnitude *E_a_* and its polarization direction γ at the same time. When we apply this spinning EO sensor method to the laser system with a femtosecond laser oscillator with an 80-MHz repetition rate, the most severe problem is that there is a large crystal-position-dependent background signal without the EO effect [76] which is so large that it may hide the EO signal with a much smaller E-field magnitude compared to the one with the regenerative amplifier. A careful design of the electronic sampling timing of the EO signals as well as the background subtraction algorithm described in [72] and [76] are both required to obtain a stable measurement.

Figure 2 shows a block diagram of the instrument electronics. A master clock frequency (*f* = 3 kHz) from the mechanical chopper is multiplied by using two phase-locked loop (PLL) electronic circuits to generate 2*f* and 32*f* clock pulses. The 32*f* clock pulses are used to determine the timings of the analog-to-digital (A/D) conversion of the Δ*I*(*T*) signal, which is stored in a personal computer (PC). The 2*f* clock pulses are used to control the hollow-shaft brushless DC motor with an encoder with 125 pulses per revolution. The rotation frequency of the motor becomes (2/125)*f* (48 Hz) which can be monitored through an electronic pulse sequence from the apparatus. The (2/125)*f* clock pulse sequence is put into a frequency divider to generate a (1/125)*f* (24 Hz) pulse sequence, which is used as a start trigger for the measurement.

A timing chart of the measurement system is shown in Figure 3. The four clock pulses are phase-matched with each other with a timing jitter of about 2 μ s, while their rising-edge timings are different. After the PC detects the start trigger pulse, another control pulse synchronized with the timing of the optical chopper initiates the sequential data acquisition of the measurement. To obtain the control pulse, we use another (∼100 kHz bandwidth; much faster detector is preferable) photo-detector and probe the time-dependent intensity profile of a part of the laser pulse after the mechanical chopper. Another technique to determine the timing of the control pulse is to maximize the modulated components of the Δ*I*(*T*) signal by changing the timing to initiate the numerical sequential data acquisition using a PC program, which is much easier since we do not need the additional photo-detector. As the frequency of the pulse used to determine the timings of the A/D conversions is exactly 32 times higher than the optical chopper frequency, the first 16 sequential data points represent the EO signal in the presence of a terahertz pulse, while the next 16 sequential data points represent those in the absence of the terahertz pulse. We numerically average these two sets of 16 sequential data points using a PC program, and call the average values the first datum (1) and the second datum (2), as shown in Figure 3. The angle between the *x*-axis and the [1̅10] direction of the rotating EO crystal is *β*_0_ when we are measuring the first datum, while it is $\left(\beta_{0}+\frac{2\pi }{125}\right)$ when we are measuring the second datum. After the recording of the 125th datum, where the crystal angle is $\left(\beta_{0}+\frac{124}{125}\times 2\pi \right)$, we continuously measure the 126th datum which is a complementary datum to the first datum, having the same angle of *β*_0_ but without the terahertz pulse due to the fractional relationship between the frequency of the DC motor and that of the optical chopper, since their ratio is 2/125 (see Figure 3). We finally obtain 250 of these averaged data values including 125 data sets with different crystal angles, and each set consists of two data values at the same crystal angle, one with and one without the terahertz pulse. For each set, we subtract one data value from the other and obtain 125 angle-dependent EO signals. The mathematical framework of the background subtraction algorithm is described in Reference [76].

## 3. Experimental Results and Discussion

**Figure 4.**
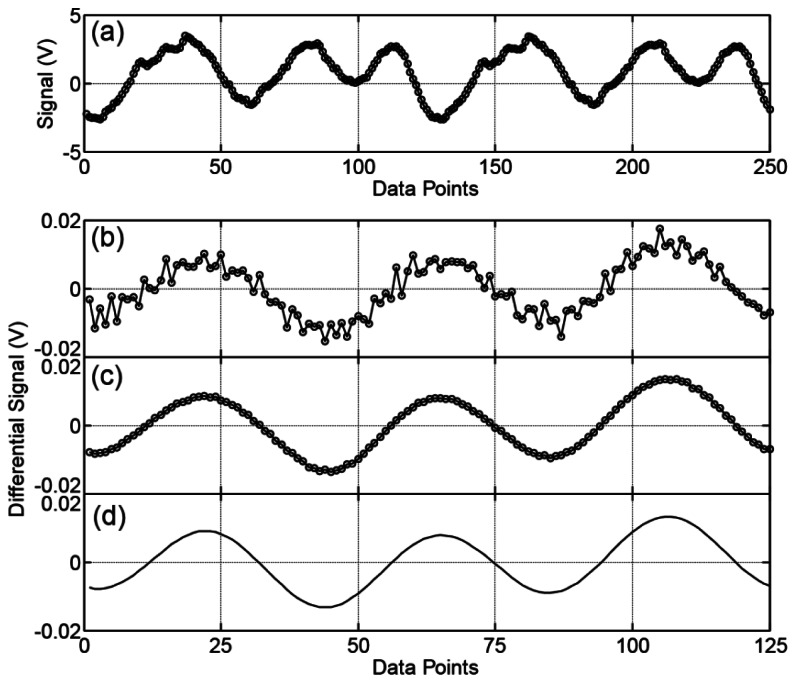
(**a**) Time-dependent intensity difference signal Δ*I*(*T*). 250 data values are obtained during two rotations of the EO sensor; (**b**), (**c**) Δ*I*(*T*) signals after we perform the background subtraction algorithm that we describe in the text. Figure 4(b) shows the experimental results for a single measurement with the two rotations of the EO crystal that finishes within 42 ms. To obtain Figure 4 (c), we perform *N* = 1,000 measurements and average the data; (**d**) A plot of the mathematical function as shown in Equation (1) when we replace the variable *T* with 2π × /(125 *ω*), where *ω* = (2π × 48) Hz is the angular rotation frequency of the EO sensor and *i* is an integer data point ranging from 1 to 125. The parameters *β*_0_ + γ = 132 [ deg. ], 3*β*_0_ − γ = 252 [deg.] are determined by a fitting of the experimental curve in Figure 4 (c).

Figure 4(a) shows the sequential 250 data values of the intensity difference signal Δ*I*(*T*) during the two rotations of the EO crystal. The voltage data ranges over ±3 V owning to the background signals that originate from the effects of the scattering of the probe laser pulse and the residual birefringence inside the EO crystal [77], which depends on the crystal rotation angles. Since we obtain 125 data values per rotation, we observe almost similar waveforms between data values 1 through 125 and data values 126 through 250. Next, we show that we can cancel this large background noise by using the background subtraction algorithm we described in the last part of the previous section. Figure 4(b,c) shows the 125 data values after we have performed the background subtraction algorithm. Figure 4(b) shows the experimental results for a single measurement that finishes within the two rotations of the EO crystal (1/(24 Hz) = 42 ms). In Figure 4(c), we perform *N* = 1,000 measurements and average the data. The voltage of the subtracted signals ranges over ±0.01 V, which is about 0.3% of the measured data with the background signals. Despite the huge difference between the magnitudes of the EO signals and the background signals, the background subtraction algorithm works effectively, and we can obtain the modulated EO data, which can be fitted by Equation (1), as shown in Figure 4(d).

**Figure 5.**
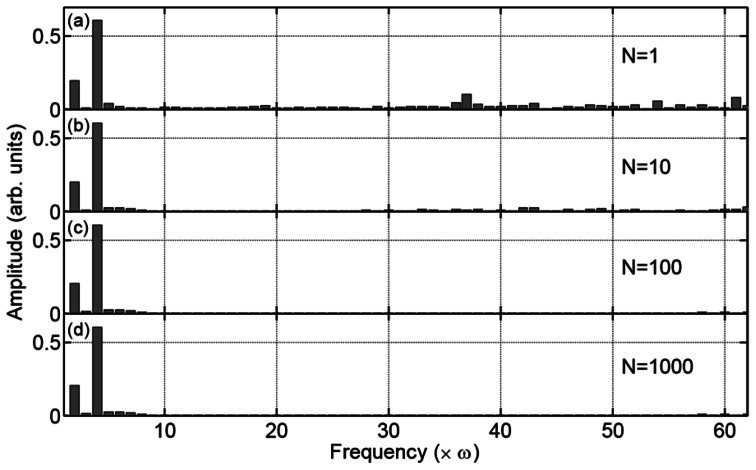
(**a**)–(**d**) Amplitude of each frequency component of the experimental data after the background subtraction for different averaging numbers *N*. We repeat the measurements (**a**) *N* = 1; (**b**) 10; (**c**) 100; and (**d**) 1,000 time(s). In the corresponding graphs, *E*_ω_/*E*_3ω_ has the value (**a**) 3.10, (**b**) 3.03, (**c**) 2.95 and (**d**) 2.95.

We perform the Fourier transform of the experimental data after the background subtraction as shown in Figure 4(b,c), and analyze the amplitude of each frequency component of the signals (*E*_nω_). Figure 5 shows the histogram of (*E*_nω_) for different averaging numbers *N*. Obviously, the amplitudes of the ω and 3 ω frequency components have the largest values with the relation *E*_3ω_ ≅ 3*E*_ω_, while there are finite amplitudes in other frequency components, which we consider to be the residual background noise. There are two kinds of residual background noise components in the data. One of the two is random in every cycle of the measurements, and thus we can eliminate the noise by averaging the experimental data by increasing the number of experiments *N*.

We believe that this noise originates from a power fluctuation of the laser pulse and/or some imperfection in the stable rotation of the motor within a single cycle of the measurement. These errors are different from experiment to experiment, and therefore, they can be cancelled by averaging the data obtained by repeatedly performing the experiment. The other kind of noise component is one that cannot be eliminated even though we average the data as we observe in the lower frequency components in Figure 5(b–d). We consider that the second noise component originates from the nonlinearity of the current-to-voltage converter in our homemade balance detector, which causes a distortion of the signal Δ*I*(*T*), and the error cannot be eliminated by averaging the data. Please note that these residual noises are, however, spectrally spread out; thus, they have less influence on the analysis of the ω and 3 ω frequency components. Therefore, a single measurement is enough to analyze the magnitude and the polarization direction of the terahertz waves despite relatively large background noise of the signals in Figure 4(b) compared to Figure 4(c).

**Figure 6.**
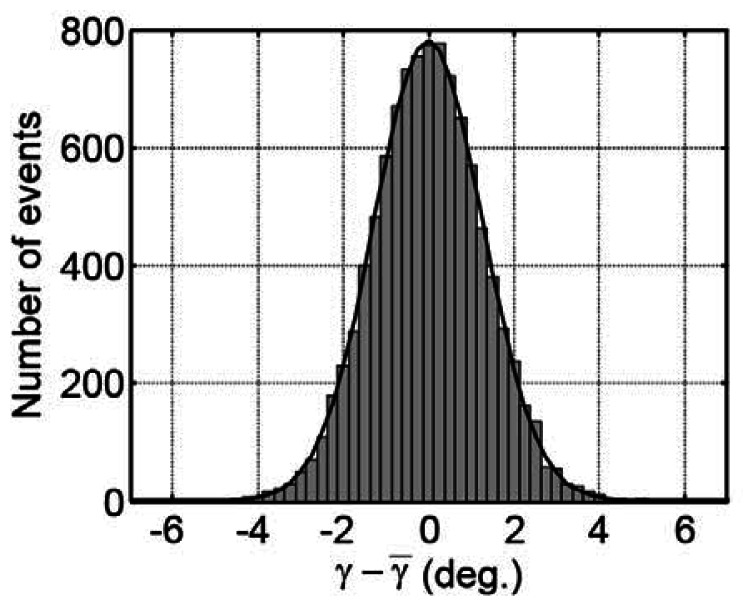
Statistical distribution of deviations of the measured polarization directions of the terahertz waves γ from the mean value γ̅ when we repeat the measurements *N* = 10,000 times. Each measurement is performed within ∼42 ms. The Gaussian fit curve (solid line) with a standard deviation of 0.95° is also shown.

We can obtain the value γ representing the polarization direction of the terahertz E-field vectors from the analysis of the phases of the ω and 3 ω frequency components using Equation (1). Figure 6 represents the statistical distribution of the measured polarization directions of the terahertz waves γ from their mean value γ̅ when we repeat the measurements *N* = 10,000 times. Each polarization direction γ is determined by the single polarization measurement that finishes within the two rotations of the EO crystal (42 ms). The standard deviation of the distributions of γ is σ=0.95°; therefore, we can determine the polarization direction of the terahertz E-field vector within about ±0.95° in 42 ms. Please note that repeating the measurements increases the precision of the estimation of the mean value γ̅. The standard error of the mean becomes $\sigma /\sqrt{N}=0.0095{}^{\circ}=0.17$ mrad in Figure 6, which is sufficient for detecting, for example, a small Kerr rotation angle to estimate the off-axis conductivity of materials [19]. Obviously, the precision to determine γ̅ depends on measurement times, and thus a long term stability of the polarization experiments is important to achieve the reliable measurements. We proved in Reference [72] that the long term intensity fluctuations of the laser pulse does not cause serious errors to determine γ, because the information on γ is retrieved from the phase information of the signal's time evolution within two cycles of the crystal rotations (∼42 ms in the present work) so that the amplitude fluctuation in time scales longer than this period does not suffer the precision to determine γ. Therefore, we can perform experiments as many times as possible to get a reliable mean value of γ. This advantage is important when we measure optically dense samples where the magnitude of the transmitted terahertz wave becomes very small and highly sensitive polarization measurements are required.

## 4. Conclusions and Outlook

In this paper, we established a real-time and precise terahertz polarization analyzer with the spinning EO sensor method by using 80-MHz repetition-rate femtosecond laser pulses. It was necessary to pay special attention needed to be paid to the electronic part of the measurement system to improve the signal-to-noise ratio. Finally, we obtained the polarization angle of the terahertz E-field vectors with respect to the polarization direction of the probe laser pulse within a measurement time of 42 ms with a standard deviation of σ=0.95°, which is comparable to those made by using a regenerative amplifier laser system. As the long term intensity fluctuations of the laser pulse does not cause serious errors to determine the polarization direction γ, we can repeat the experiments to obtain a reliable mean value (γ̅) of γ. This success promises to realize a broadband and compact real-time terahertz time-domain polarization measurement system that would be useful for scientific and industrial applications.
